# Comparative Population Genomics of African Montane Forest Mammals Support Population Persistence across a Climatic Gradient and Quaternary Climatic Cycles

**DOI:** 10.1371/journal.pone.0131800

**Published:** 2015-09-22

**Authors:** Terrence C. Demos, Julian C. Kerbis Peterhans, Tyler A. Joseph, John D. Robinson, Bernard Agwanda, Michael J. Hickerson

**Affiliations:** 1 Biology Department, City College of New York, City University of New York, New York, New York, 10031, United States of America; 2 Biology Doctoral Program, The Graduate School and University Center, City University of New York, New York, New York, 10016, United States of America; 3 Integrative Research Center, Field Museum of Natural History, Chicago, Illinois, 60605, United States of America; 4 College of Professional Studies, Roosevelt University, Chicago, Illinois, 60605, United States of America; 5 South Carolina Department of Natural Resources, Charleston, South Carolina, 29412, United States of America; 6 Mammalogy Section, National Museums of Kenya, Nairobi, 00100, Kenya; 7 Division of Invertebrate Zoology, American Museum of Natural History, New York, New York 10024, United States of America; University of Colorado, UNITED STATES

## Abstract

The Eastern Afromontane biodiversity hotspot (EABH) has the highest concentration of biodiversity in tropical Africa, yet few studies have investigated recent historical diversification processes in EABH lineages. Herein, we analyze restriction-site associated DNA-sequences (RAD-Seq) to study recent historical processes in co-distributed mouse (*Hylomyscus*) and shrew (*Sylvisorex*) species complexes, with an aim to better determine how historical paleoenvironmental processes might have contributed to the EABH’s high diversity. We analyzed complete SNP matrices of > 50,000 RAD loci to delineate populations, reconstruct the history of isolation and admixture, and discover geographic patterns of genetic partitioning. These analyses demonstrate that persistently unsuitable habitat may have isolated multiple populations distributed across montane habitat islands in the Itombwe Massif and Albertine Rift to the west as well as Mt Elgon and Kenyan Highlands to the east. We detected low genetic diversity in Kenyan Highland populations of both genera, consistent with smaller historical population sizes in this region. We additionally tested predictions that Albertine Rift populations are older and more persistently isolated compared to the Kenyan Highlands. Phylogenetic analyses support greater historical isolation among Albertine Rift populations of both shrews and mice compared to the Kenyan Highlands and suggest that there are genetically isolated populations from both focal genera in the Itombwe Massif, Democratic Republic of Congo. The Albertine Rift ecoregion has the highest mammalian tropical forest species richness per unit area on earth. Our results clearly support accelerating efforts to conserve this diversity.

## Introduction

**Fig 1:**
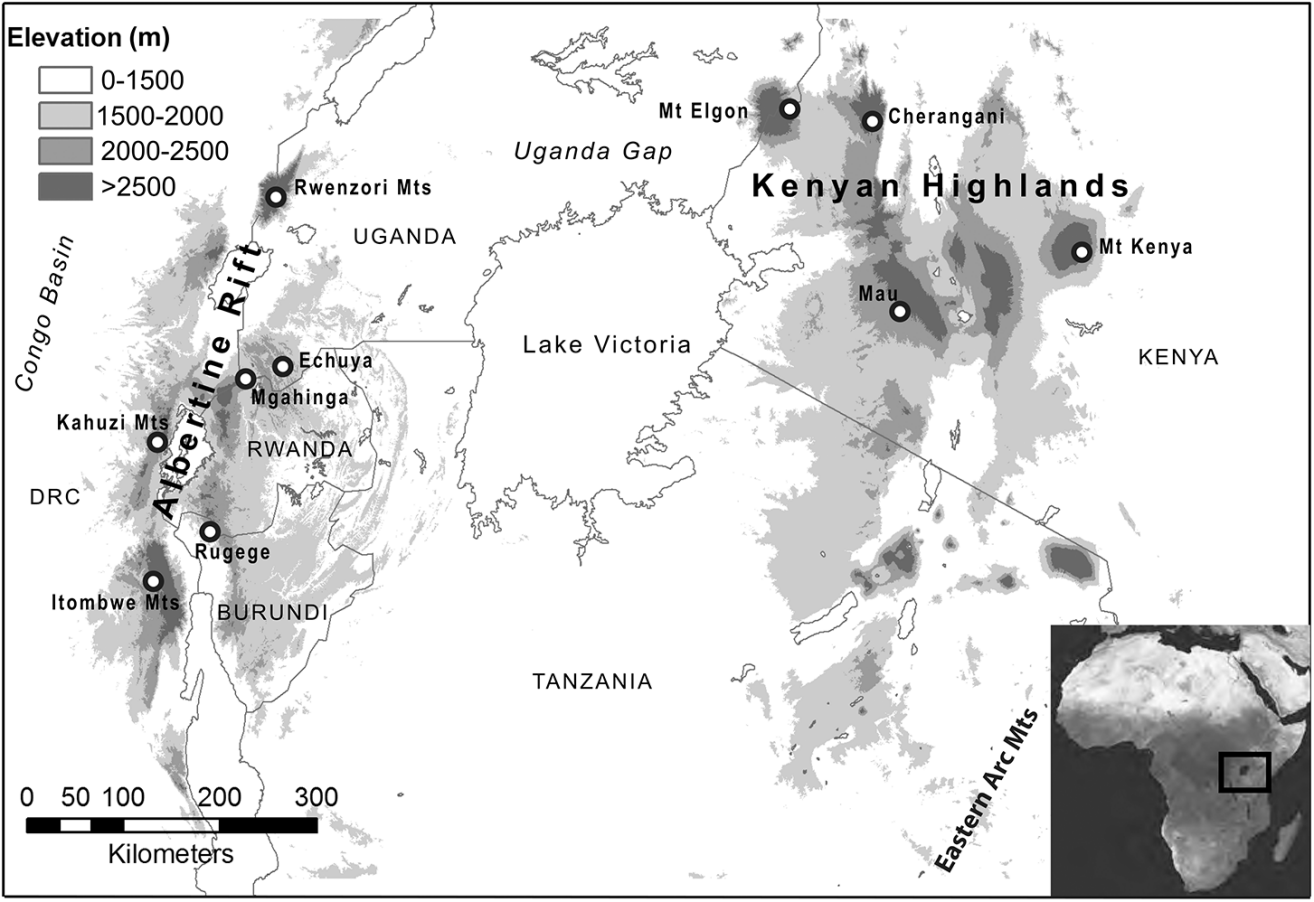
Map of sample collection sites in the Eastern Afromontane region. Collection localities are described in S1 Appendix. Sampling locality abbreviations are as follows: Albertine Rift; Ruwenzori Mts (RUW), Echuya (ECH), Mgahinga (MGA), Kahuzi Mts (KAH), Rugege (RUG), Itombwe Mts (ITO); Kenyan Highlands; Mt Elgon (MTE), Cherangani (CHE), Mt Kenya (MTK).

Understanding the evolutionary processes that generate and maintain biodiversity is essential for identifying regions of global conservation importance [1,2]. Over the last decade, considerable progress has been made in delimiting and categorizing global biodiversity hotspots based on species richness and endemism. However, there is still a critical need to quantify genetic structure within species to better understand fine-scale geographic patterns of biodiversity. In particular, the Eastern Afromontane biodiversity hotspot (EABH) encompassing the Albertine Rift Mts (AR), Kenya Highlands (KH) and Eastern Arc Mts of east central Africa has been understudied in this regard. The EABH holds perhaps the greatest concentration of mammalian diversity on earth per unit area [3] (Fig 1). Indeed, recent taxonomic and phylogeographic research has uncovered previously unknown species and multiple genetically divergent, but morphologically cryptic lineages [4–9].

Recently, the first mammalian multi-locus comparative phylogeographic study of the EABH supported the persistence of currently disjunct shrew and rodent populations over multiple Pleistocene climatic cycles. This same study also uncovered morphologically cryptic diversity in several *Sylvisorex* shrew and *Hylomyscus* mouse lineages [6]. Demos et al. (2014) presented evidence showing distinct patterns of regional sub-division and demography across focal taxa. They attributed differences to variation in life-history traits, while explaining similarities as shared responses to regional variation along an east-west climatic gradient (i.e. more mesic AR vs. more xeric KH). In addition, the authors used Bayesian coalescent species delimitation [10] with 2–3 nuclear loci/focal taxon, and identified strong support for multiple evolutionarily significant, previously unrecognized lineages (putative species).

Due to the previous study’s limited genetic sampling (3–6 loci), we regard these findings as provisional and in need of further testing [11,12]. Population structure and natural selection often vary across the genome [13–16], such that broad sampling is necessary for confident and precise population genetic inference. Accurate population assignment is particularly difficult in the tropics where taxonomic resolution is often poor [17,18], particularly among recently diverged and morphologically cryptic taxa [19]. This problem is pervasive even within well-studied vertebrate groups such as mammals, where new species are regularly described [20]. Obtaining population-level genomic sampling is therefore an essential tool for mitigating these difficulties.

Here we use high-throughput-sequencing to investigate genome-wide patterns of population structure and gene flow within and between two co-distributed EABH montane-endemic species complexes; the *Hylomyscus denniae* complex (African montane wood mouse) and the *Sylvisorex granti* complex (Grant’s forest shrew). Within the *H*. *denniae* complex we obtained RAD-Seq genomic data from *H*. *denniae*, *H*. *vulcanorum*, *H*. *endorobae* and *H*. *kerbispeterhansi* individuals that were sampled from 8 mountain ranges (populations) across the Albertine Rift and Kenya Highlands (Fig 1). In addition, data within the *S*. *granti* complex from the *S*. *granti*, *S*. *vulcanorum* and *S*. *mundus* members of the complex were obtained from the same 8 mountain ranges and two additional mountain ranges. The resulting data (56,194 SNPs in *Hylomyscus* and 52,580 SNPs in *Sylvisorex* in complete matrices) allow us to explore Pleistocene refugial population structure and pulses of admixture and characterize genetic variation across the entire range of two co-distributed African montane endemics. Although we base inference from 1–6 individuals per each of 10 localities, data from large numbers of unlinked loci allow highly resolved inference even with few individuals [11,21]. Furthermore, genome-level datasets should capture the diversity of coalescent histories (across loci) that reflect population history, such that information comes more from the number of loci sampled through the genome than from numbers of individuals per sampling locality [22–24].

Several aspects of the geological and climatic history of the EABH lead to straightforward expectations for biodiversity patterns. First, both paleo-climatic and geological data from many studies indicate that the Albertine Rift is older, more topographically complex, and more climatically stable than the Kenya Highlands to the east [25,26]. It is also well supported that pan-African forests underwent fragmentation during a series of repeated aridification events peaking at 2.8, 1.7 and 1.0 Ma [27,28] and that intensification of glacial cycles during the late-middle Pleistocene generated more extreme fragmentation of tropical forests in Africa. Reductions in forest cover during the Last Glacial Maximum (LGM) [29] are also thought to have been more pronounced in the KH relative to central Africa and the AR. These factors lead to the testable hypothesis that the AR had a longer history of local isolation and persistence at the population and species level than the KH. This would predict that (1) genetic diversity will be lower in KH populations than in AR populations, and (2) the AR having greater population structure and harboring more undescribed phylogenetic diversity than the KH.

To test this hypothesis, we use high-throughput genome-wide sequencing to estimate historical demographic parameters underlying relative isolation times, historical effective population sizes, levels of population structure and post-divergence gene flow within and between two co-distributed EABH montane-endemic species complexes: the *Hylomyscus denniae* complex (African montane wood mouse) and the *Sylvisorex granti* complex (Grant’s forest shrew). The combination of broad geographic sampling in this study and deep population sampling made possible by genome-enabled phylogeographic data enable a more biogeographically informative and better supported evolutionary history of these species groups that offer significant departures from earlier studies.

## Materials and Methods

### Sample collection and DNA extraction

We sampled populations of *Hylomyscus* and *Sylvisorex* from 8 and 9 montane blocks across the Eastern Afromontane region, respectively. Within the AR and KH, members of the *H*. *denniae* and *S*. *granti* complexes are co-distributed on every mountain. We collected 2–4 individuals of each genus on 7 mountain ranges where they co-occur. All specimens and tissue samples were deposited in the Field Museum of Natural History (FMNH) under the approval of the FMNH Institutional Animal Care and Use Committee (S1 Appendix). Permit authorization for specimen collection, export, and subsequent deposition at FMNH was issued by the following agencies: Burundi, Institut National pour l'Environment et la Conservacion de la Nature; Democratic Republic of the Congo, Institut Nacional pour la Conservacion de la Nature and Centre de Recherché des Sciences Naturelles; Kenya, Kenya Wildlife Service, Kenya Forest Service and National Museums of Kenya; Rwanda, Rwanda Development Board, National University of Rwanda and Ministry of Natural Resources (MINIRENA); Uganda, Uganda Game and Fisheries Department and Uganda Wildlife Authority. None of the specimens included in this study are listed under CITES protection appendices. We isolated genomic DNA from 20 mg of muscle tissue using a DNeasy Blood and Tissue kit (Qiagen) after an initial digestion with 4 μl of 100 μM RNAse A. DNA samples were quantified using Qubit Broad-Range Fluorometric Quantitation (Invitrogen, Carlsbad, CA) and genomic DNA quality was checked by visual inspection of an agarose gel.

### RAD library preparation and SNP genotyping

Ten micrograms of high molecular weight genomic DNA per individual was submitted to Floragenex Inc. (Eugene, OR) for library preparation and high-throughput sequencing following the methods outlined in Baird et al. [30], Hohenlohe et al. [31] and Emerson et al. [32]. Individual sample specific identifying barcodes and sequencing adaptors were ligated to total genomic DNA that was digested using a high-fidelity SbfI restriction enzyme. Five base pair sequence tags were added as unique barcodes. The resulting fragments were multiplexed and sequenced on an Illumina HiSeq 2000 platform using single-end 100 bp reads. Samples were de-multiplexed, separated by individual, and barcode sequences removed, leaving RAD tags of 90 basepairs. Sequences were aligned to *de novo* RAD pseudo-reference genomes for each genus.

The two RAD references came from a single individual per genus after preliminary analysis identified which had the greatest number of unique RAD tag clusters. Custom perl scripts were used to group identical sequences represented by 5 to 500x coverage. Reads for each individual were aligned to the RAD pseudo-reference genome using BOWTIE [33] using sequence quality information that allowed a maximum of three mismatches. Alignment to no more than one reference region per read was allowed. SAMTOOLS algorithms [34] and custom Floragenex scripts were used to detect SNPs and determine genotypes. Initial filters for genotyping required a minimum Phred quality score of 15, a minimum of 4x, 6x, or 10x sequence coverage, and a minimum of 65–85% of the population genotyped across samples. The different sequence coverage cutoffs provided three different genotyped data sets that were tabulated and formatted in the variant call format [35]. We note that individuals lacking restriction enzyme recognition sites on either chromosome as a result of polymorphisms will not be targeted by a given enzyme. Because of this the RAD-Seq method may result in downwardly biased estimates of polymorphism [36]. However, Davey et al. [37] showed that if sufficient depth of sequencing coverage is achieved, coupled with conservative filtering criteria (we used q > 20) then RAD loci present no biases that are not already widely known.

### Data quality and coverage filtering

We used the relaxed filter applied by Floragenex to call genotypes (*i*.*e*. q > 15, 4x coverage, and 65% population coverage) and applied our own custom filtering pipeline to minimize both false positive genotype detection of heterozygotes and the impact of paralogous regions. VCFtools was used to retain only those SNPs for which a minimum and maximum of 2 alleles were present. To ensure coverage adequate for correctly evaluating heterozygous sites and control for paralogous sequence variants [38], all SNPs with < 8x coverage and Phred scores < q 20 were removed, as were those with coverage ≥ 1.5 standard deviations above the mean coverage across all RAD tags (77x coverage for mouse, 108x coverage for shrew). For a subset of analyses, an additional set of filters was used to generate complete matrices that contained one random SNP per RAD locus for both genera at the species complex and individual species levels. Only bi-allelic SNPs were included in order to simplify calculations and fit the assumptions of software used to analyze these data (see below).

### SNP analysis

**Fig 2:**
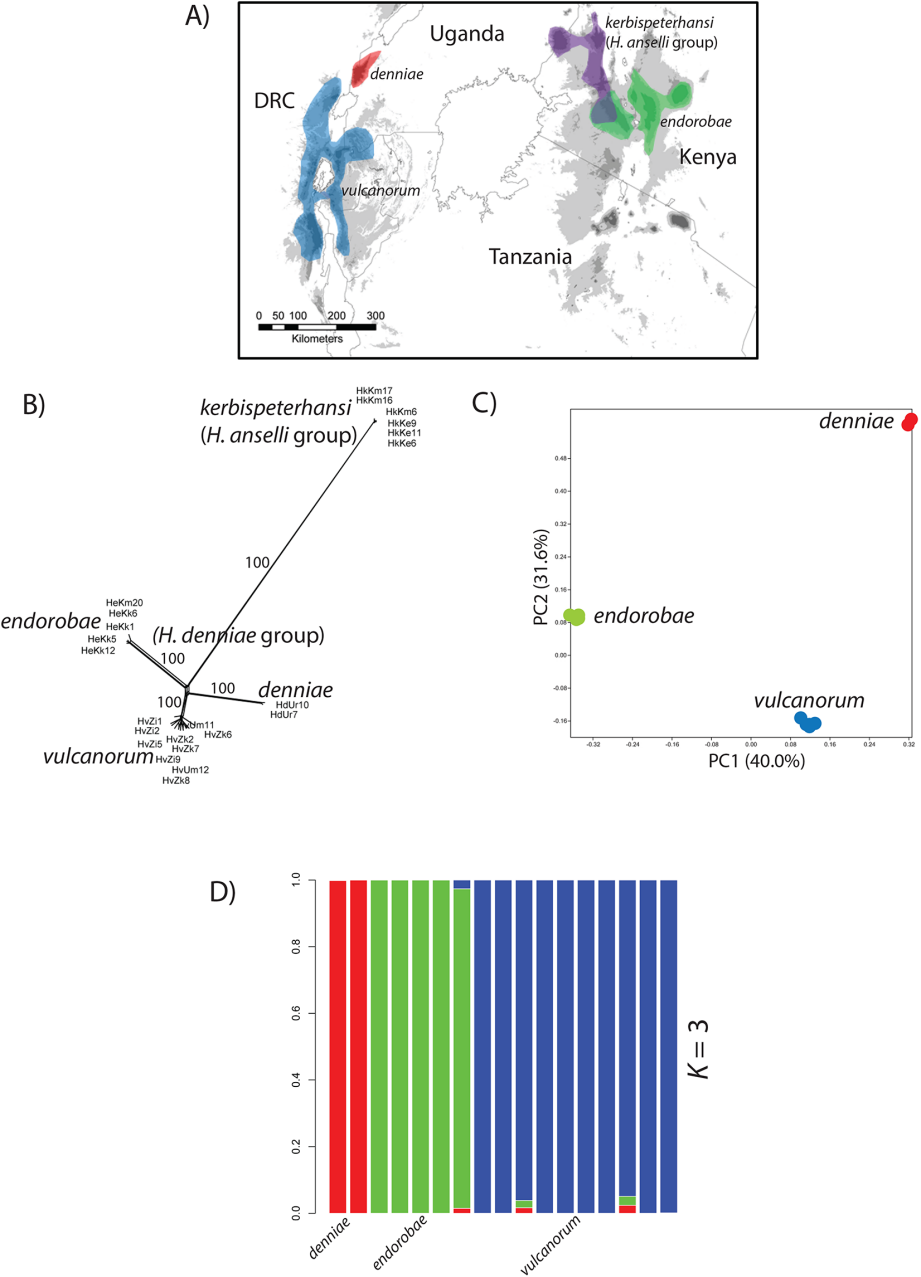
Distribution of focal taxa and results of exploratory population delimitation analyses for *Hylomyscus* focal species. (A) Geographic distribution of study taxa within the *H*. *denniae* and *H*. *anselli* groups. (B) Network graph inferred in SplitsTree using the Neighbor-Net algorithm for species within the *H*. *denniae* and *H*. *anselli* groups with bootstrap proportions indicated for major nodes. (C) PCA of individuals within the *H*. *denniae* group. (D) Estimates of admixture proportions inferred with sNMF for *H*. *denniae* group species under the best-supported number of populations.

**Fig 3:**
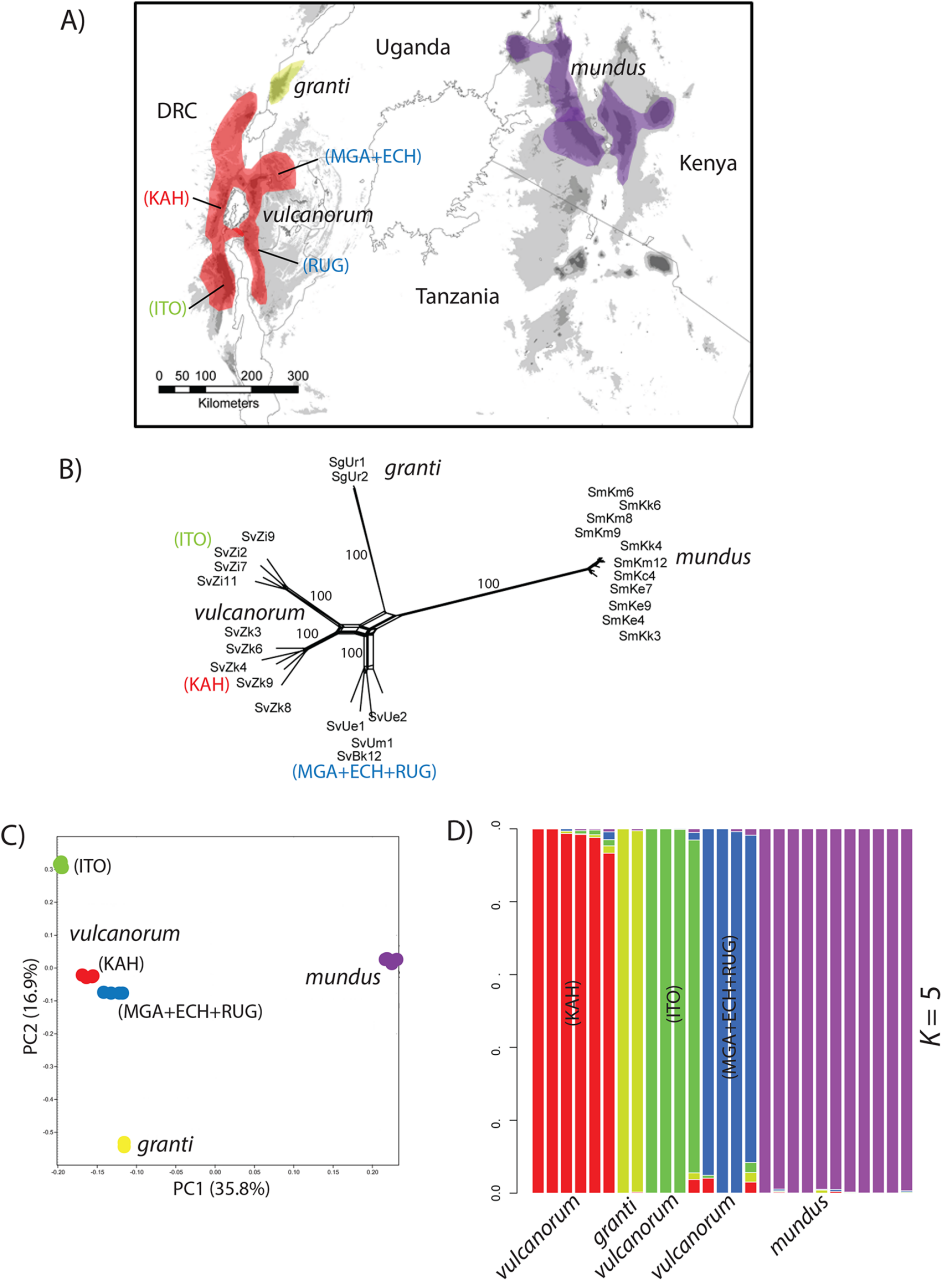
Distribution of focal taxa and results of exploratory population delimitation analyses for *Sylvisorex* focal species. (A) Geographic distribution of study taxa within the *S*. *granti* group. (B) Network graph inferred in SplitsTree using the Neighbor-Net algorithm for species within the *S*. *granti* group with bootstrap proportions indicated for major nodes. C) PCA of individuals within the *S*. *granti* group. (D) Estimates of admixture proportions inferred with sNMF for *S*. *granti* group species under the best-supported number of populations.

We sampled 1–4 individuals per locale from the *Hylomyscus denniae* group (*H*. *denniae*, *H*. *endorobae* and *H*. *vulcanorum*) and the *Sylvisorex granti* species group (*S*. *granti*, *S*. *vulcanorum* and *S*. *mundus*). Two closely related outgroups per species group were also sampled. Within Kenya, a newly described species of *Hylomyscus* from the *Hylomyscus anselli* species group is co-distributed with *Sylvisorex* in western Kenya [7]. To compare population structure and demography in this region, we also sampled two populations of this group. Both shrew and mouse samples were obtained from across the entire geographic distribution of both species groups and, when possible, where both species are known to be co-distributed. Mice and shrews were sampled from a total of 8 and 9 montane blocks, respectively (Fig 1). Within the AR and KH, the *H*. *denniae* and *S*. *granti* complexes are co-distributed on the majority of mountain ranges sampled to date (Figs 2 & 3).

### Population genetic analyses

Summary statistics (*H*_*e*,_ Weir & Cockerham’s *F*_*st*_ *)* were estimated using components of VCFtools v4.0 package. To visually explore how many populations the samples were derived from and how best to assign individual genotypes from these populations, we implemented a principal component analysis (PCA) using the software SmartPCA [39] in EIGENSOFT v4.2. PCA was performed with default setting for both focal species groups. Because missing data can bias inference of population structure if the missing data are structured [39] we used only complete SNP matrices (*Hylomyscus denniae* complex and species therein *n* = 56,194; *Sylvisorex granti* complex and species therein, *n* = 52,580).

We further examined population structure and assignment of genotypes to populations using the population clustering method implemented in sNMF [40], a new method for estimating individual ancestry coefficients that accommodates large population genomic data sets through the use of an efficient likelihood algorithm without loss of accuracy. Briefly, this method considers an individual’s diploid genome as from a specified number (*K*) of hypothetical ancestral populations and then infers the proportion of each individual’s genome derived from these ancestral populations. We estimated ancestry coefficients across a range of *K* values equal to the number of sampled mountain ranges plus one (1–11 for the *Sylvisorex* group and 1–8 for the *Hylomyscus* group). Separate analyses were carried out for individual species distributed on two or more montane blocks across a range of *K* values. Ten replicates were run for each value of *K* for both the inter- and intra-specific data sets. Cluster membership coefficients were identified using the cross-entropy criterion implemented in sNMF based on the prediction of masked genotypes (5% of the data set). This was done to evaluate the error of ancestry estimation and guide choice of the number of ancestral populations. Variance among runs of the cross-entropy statistic was evaluated by carrying out 10 replicates for every *K* value. sNMF results were visualized in R [41] across a range of *K* values. To further visualize population structure, we used the Neighbor-Net algorithm [42] implemented in the program SplitsTree v4.13 [43] to generate phylogenetic networks of alleles. Support levels for inferred splits were assessed using 1000 bootstrap replicates.

To find the population graph that best described the relationships among populations in the datasets, we used the software TreeMix [44] that indicates pairs of populations that are candidates for admixture events. This method uses genomic-scale allele frequency data to find the best-supported maximum-likelihood tree for a set of populations and then infers admixture (migration events) through identification of populations that have a poor fit to the tree. Divergence is represented as nodes in the resulting maximum likelihood tree, branch lengths are proportional to divergence time and the degree of genetic drift experienced by populations. Migration events are represented by edges that connect populations with admixture from ancestral populations. We first generated a maximum-likelihood graph with no migration events and then tested for a range of migration events (*n* = 1–6 within *Hylomyscus*; *n* = 1–10 within *Sylvisorex*) including possible admixture with the closest known outgroup in both analyses. The best-supported number of migration events was inferred by dividing the residual covariance between each pair of populations, *i* and *j*, by the average standard error across all pairs. Because the TreeMix model is heavily parameterized, the program author’s caution against placing strict confidence on *p-*values generated from the Wald statistic. Instead, they recommend implementation of the three-population test using the *f*_3_ statistic [45] and the four-population test using the *f*_4_ statistic. These tests were carried out and compared with the Wald migration edge *p*-value.

We estimated population relationships from SNPs in SNAPP (part of BEAST 2.0) which is software used to infer population trees and demography from independent bi-allelic markers [46]. This package implements a full coalescent model to integrate over all possible gene trees rather than sampling them explicitly. SNAPP was run for at least 1,000,000 generations with sampling every 1000 generations. Posterior parameter convergence was assessed using Tracer version 1.5 (http://tree.bio.ed.ac.uk/software/tracer/). All parameters had effective sample size (ESS) values > 100. Convergence of the posterior topology was assessed visually from trace plots. After removal of the first 20% of sampled trees as burn-in, maximum clade credibility trees were generated with TreeAnnotator using BEAST 2.0. No estimate of nuclear mutation rate is available for *Hylomyscus or Sylvisorex*, so we used the mutation rate 1.0 × 10^−8^ per year estimated from divergence in mammals [47,48] and assumed a generation time of one year.

To test whether patterns of shared polymorphism between AR and KH populations are due to admixture, ancestral structure, or incomplete lineage sorting in ancestral panmictic populations, we implemented the maximum likelihood method described in Lohse et al. [22] and Lohse & Frantz [49]. We calculated likelihood values under six models of admixture varying in direction, an ancestral structure model, and a model of strict divergence without gene flow with panmictic ancestral populations. Briefly, the Lohse method calculates the maximum likelihood of a particular model by counting the shared derived mutations of short blocks of DNA, in this case for each RAD tag. Since this method can allow for > 1 SNP per block, we used pre-filtered data to include all SNPs per block. Furthermore, because the method assumes three populations with one haploid individual per population, we randomly picked one haploid per population for triplets based on a three-population phylogeny suggested from the PCA, sNMF and SNAPP analyses. We phased all RAD tags from each population triplet and an outgroup using the program fastPHASE, and picked a random haplotype for each population and the outgroup at each tag. We then polarized the randomly sampled SNPs at each tag using the outgroup genotype (*Sylvisorex lunaris* for *Sylvisorex*, and *Hylomyscus kerbispeterhansi* for *Hylomyscus*). Maximum likelihood analyses were carried out in Mathematica 8 using the supporting notebooks from Lohse & Franz [49] and Hearn et al. [24]. We assessed statistical support between models with a likelihood ratio test.

## Results

### SNP data quality and processing

We obtained 143,726,000 single-end Illumina reads of 90 bp length from 28 individuals within *Hylomyscus* and 238,291,000 single-end reads from 31 individuals within *Sylvisorex*. We extracted 56,194 SNPs from 20,021 RAD loci shared by all individuals in the *Hylomyscus denniae* complex and 52,580 SNPs from 18,658 RAD loci shared by all members of the *Sylvisorex granti* complex. With outgroups included, there were 47,427 and 18,385 SNPs in the *Hylomyscus* and *Sylvisorex* data sets, respectively.

### Population assignment and delineation

To aid in the determination of population units for downstream inference of demographic history, we used phylogenetic network inference [43], principle component analysis [39], and population clustering based on ancestry coefficients [40]. The Neighbor-Net algorithm in SplitsTree recovered phylogenetic clustering for *Hylomyscus* consistent with current taxonomy at the species level (Fig 2). Within the focal *Sylvisorex* species group, 3 distinct clades are inferred for *S*. *vulcanorum* from the Albertine Rift along with separate clusters for *S*. *granti* and *S*. *mundus* (Fig 3). These groupings for *S*. *vulcanorum* are not recognized in the current taxonomy.

**Fig 4:**
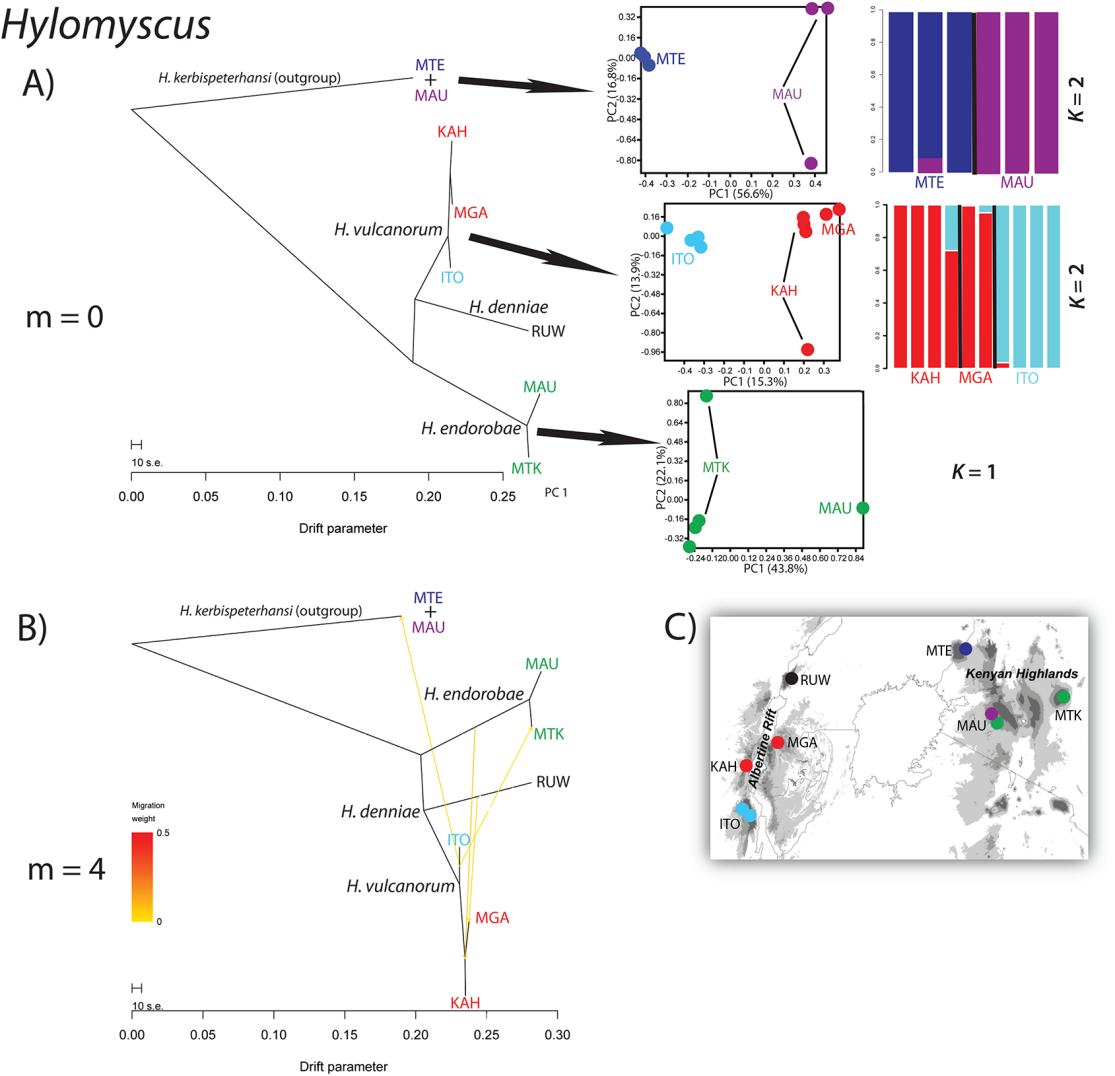
Results of the TreeMix, PCA and sNMF analyses for populations in three species in the *Hylomyscus denniae* group. (A) The maximum-likelihood tree inferred with TreeMix depicting phylogenetic relationships of 6 populations in three species of *Hylomyscus* and an outgroup. To the right are PCAs and sNMF estimations of admixture proportions for three species of *Hylomyscus*. (B) Population graph that depicts phylogenetic relationships and the best-supported number of admixture events (m). (C) Map depicting sampling localities used in the analyses.

**Fig 5:**
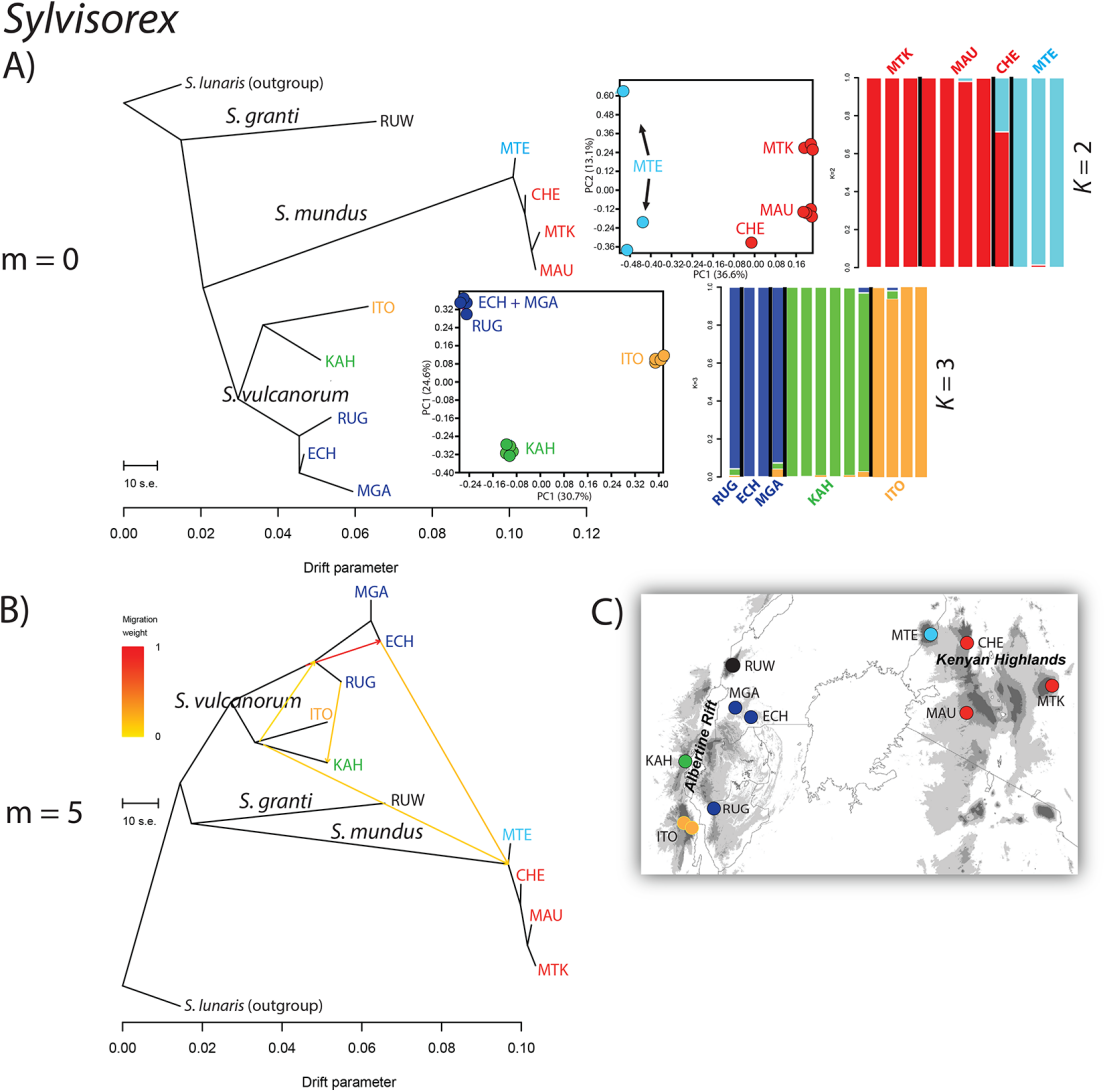
Results of the TreeMix, PCA and sNMF analyses for populations in three species in the *Sylvisorex granti* group. (A) The maximum-likelihood tree inferred with TreeMix depicting phylogenetic relationships of 10 populations in three species of *Sylvisorex* and an outgroup. To the right are PCAs and sNMF estimations of admixture proportions for *S*. *mundus* (above), *and S*. *vulcanorum* (below). (B) Population graph that depicts phylogenetic relationships and the best inferred number of admixture events (m). (C) Map depicting sampling localities used in the analyses.

The PCA conducted on SNP genotypes for both species complexes at the interspecific level showed 3 major clusters within *Hylomyscus* (Fig 2) and 5 major clusters in *Sylvisorex* (Fig 3). In the *Hylomyscus denniae* complex as a whole, PC1 separates *H*. *vulcanorum* individuals from *H*. *endorobae* individuals while PC2 separates these two complexes from individuals of *H*. *endorobae* (Fig 2). In *Sylvisorex* PCs 1 and 2 contribute almost equally to separating *S*. *vulcanorum*, *S*. *mundus* and *S*. *granti* (Fig 3). At the intraspecific level, within the AR, PC2 clearly separates Itombwe populations of both *H*. *vulcanorum* and *S*. *vulcanorum* from all other AR populations (Figs 4 & 5). In *Hylomyscus* the other two AR populations from Kahuzi and Mgahinga show little differentiation aside from one outlier individual, while in *Sylvisorex* PC1 strongly separates Kahuzi from Mgahinga + Echuya + Rugege. Within the KH, PC2 clearly separates Mau and Mt Elgon populations *of H*. *kerbispeterhansi*, while PC2 widely separates the individuals within the Mau population from one another. A similar pattern is uncovered for *S*. *mundus* where PC2 shows differences between Mt Elgon with the three mountain ranges to the east. As with *Hylomyscus*, PC1 clearly separates *Sylvisorex* individuals from the Mt Elgon population into two different clusters (Fig 5).

As a further exploratory analysis of potential population structure and delineation, we analyzed complete genotype matrices for both species complexes using the clustering algorithm sNMF [40]. To avoid potential problems with strong LD for multiple SNPs within a 90 bp RAD locus, we randomly sampled one SNP per RAD tag. Using the cross-entropy method in sMNF, we found that *K* = 3 best fit the data for the *Hylomyscus denniae* complex in a combined interspecific analysis based on current taxonomy (Fig 2). This supports recent taxonomic revisions and phylogenetic analyses for this group [7,50]. Within the *Sylvisorex granti* complex, *K* = 5 was best supported (Fig 3), others (*K* = 2–5) received only slightly less support. *Sylvisorex granti* and *S*. *mundus* were supported as independent while *S*. *vulcanorum* was grouped into 3 clusters representing populations from Itombwe, Kahuzi, and three combined populations from eastern AR. Population clustering analyses at the intraspecific level within *Sylvisorex* support *K* = 3 for *S*. *vulcanorum* and *K* = 2 for *S*. *mundus*; they are congruent with the inferences made from the population PCA data (Fig 3). Within *Hylomyscus*, the sNMF clustering algorithm supports *K* = 2 for *H*. *vulcanorum*. This is also in accordance with the within species-level PCA along axis 1 (Fig 2). Both PCA and sNMF analyses support *K* = 2 for *H*. *kerbispeterhansi* populations from western Kenya. Within *H*. *endorobae*, examination of admixture histograms revealed genetic structure at several values of *K*, however, replicate sNMF runs show that assignment of individuals to clusters was random across sNMF runs.

### Genetic diversity and population differentiation

For all loci that were polymorphic in at least one population in which > 2 individuals were sampled, the average observed heterozygosity (*H*_*obs*_) ranged from 0.005 to 0.123 in *Hylomyscus* and from 0.008 to 0.090 in *Sylvisorex* (Table 1). Kenyan Highland populations contained markedly less genetic diversity relative to Albertine Rift populations for both species complexes. For species restricted to the KH, *H*_*obs*_ was 0.008, 0.033, and 0.012 for *H*. *kerbispeterhansi*, *H*. *endorobae and S*. *mundus*, respectively. In comparison, *H*_*obs*_ for the AR restricted *H*. *vulcanorum* and *S*. *vulcanorum* was 0.120 and 0.086, respectively. Overall, AR populations had approximately 7 times the genetic diversity of KH populations, as measured by observed heterozygosity.

**Table 1:** Genome-wide heterozygosity for populations of *Hylomyscus* and *Sylvisorex*.

Region	Population	Species	*n*	*H*_*obs*_
Albertine Rift	Itombwe	*H*. *vulcanorum*	4	0.122
	Itombwe	*S*. *vulcanorum*	4	0.085
	Kahuzi	*H*. *vulcanorum*	4	0.115
	Kahuzi	*S*. *vulcanorum*	6	0.083
	Mgahinga	*H*. *vulcanorum*	2	0.123
	Mgahinga + Echuya	*S*. *vulcanorum*	3	0.090
Kenyan Highlands	Mt Kenya	*H*. *endorobae*	4	0.037
	Mt Kenya	*S*. *mundus*	3	0.008
	Mau	*H*. *kerbispeterhansi*	3	0.005
	Mau	*S*. *mundus*	4	0.009
	Mt Elgon	*H*. *kerbispeterhansi*	3	0.011
	Mt Elgon	*S*. *mundus*	3	0.018

Populations from the western side of the AR (Kahuzi and Itombwe) and across the AR valley (Mgahinga and Kahuzi) grouped together for *Hylomyscus* (*F*_*st*_ = 0.025 and 0.005, respectively) but were highly diverged from each other among three populations of *Sylvisorex* (*F*_*st*_ = 0.263–0.332; Table 2). Within the KH, *H*. *kerbispterhansi* populations from Mau and Mt Elgon both strongly differentiated from one another (*F*_*st*_ = 0.351) while in *S*. *mundus* all KH populations were notably divergent from other populations in pairwise comparisons (i.e. *F*_*st*_ = 0.184–0.310).

**Table 2:** Pairwise comparison of genetic distance (*F*_ST_) among *Hylomyscus* and *Sylvisorex* populations.

*Species*	*F*_ST_	
***H*. *vulcanorum***	Mgahinga	Kahuzi-Biega
Mgahinga		
Kahuzi-Biega	0.005	
Itombwe	0.043	0.025
***H*. *kerbispeterhansi***	Mau	
Mt Elgon	0.351	
***S*. *vulcanorum***	Kahuzi-Biega	Itombwe
Kahuzi-Biega		
Itombwe	0.295	
Echuya	0.263	0.332
***S*. *mundus***	Mt Kenya	Mau
Mt Kenya		
Mau	0.184	
Mt Elgon	0.310	0.295

### Inference of population divergence and admixture

Patterns of population divergence and admixture were inferred with TreeMix v1.12 [44] in order to find the population graph that best describes the relationships among populations while allowing for gene flow after divergence. To group individuals into taxonomic units for TreeMix, we used our PCA results and the sNMF clustering algorithm. Seven maximum likelihood trees (m = 0–6) were inferred for populations within *Hylomyscus* and 11 trees (m = 0–10) inferred for populations within *Sylvisorex*. These population graphs are consistent with population relationships found in previous phylogenetic analyses [6], with some notable exceptions. Within *Hylomyscus*, the *H*. *denniae* population was consistently recovered as sister to *H*. *vulcanorum*, whereas previous species tree analyses at this node were unresolved. Analysis of residuals for m = 0–6 showed that the graph that best fit the data inferred four interspecific migration events, the largest one from the ancestor of central Kenyan *H*. *endorobae* (Mt Kenya + Mau) to the ancestor of *H*. *vulcanorum* populations from Kahuzi + Mgahinga that had a weight (i.e., proportion of ancestry inferred from origin population) of 8%; three additional interspecific migration events had weights between 1–4% (Fig 4).

Within *Sylvisorex vulcanorum* there is a clear split between populations on the western side of the AR and those on the eastern side, but an additional split is apparent between the two western populations from Itombwe and Kahuzi. Analysis of residuals best supported an inferred graph with 5 migration events (Fig 5). The admixture proportions for these events were much higher than among populations within *Hylomyscus*. However, the most striking admixture event infers that the ancestor of all 4 populations of KH *S*. *mundus* trace 16.5% of their ancestry to an admixture event from *S*. *vulcanorum* from Echuya on the eastern side of the AR and 12% of their ancestry to an admixture event with *S*. *vulcanorum* from Kahuzi on the western side of the AR. The three remaining admixture events (8–89%) are among *S*. *vulcanorum* populations from the AR (Fig 5).

To further examine these migration events we next used three and four population tests [45,51] to statistically evaluate support for these instances of historic admixture pulses. Within *Hylomyscus* populations 4 inferred admixture events were evaluated using *f*4 and *f*3 statistics with a weighted block jackknife approach. Results for all 4 admixture events failed the *f*4 and *f*3 tests with *Z* scores of 0.0–19 and associated *P-*values of 0.023–5 x 10^−5^. Failure of *f*4 or *f*3 tests indicate that results are consistent with admixture as a result of a migration event, although there is uncertainty in precise migration estimates. Within *Sylvisorex*, *f*4 and *f*3 tests supported all five instances of admixture under the best-supported residual model. Support for these five migration events ranged from *Z* = 0.46–17 (*P* = 0.0002–0.0018).

**Fig 6:**
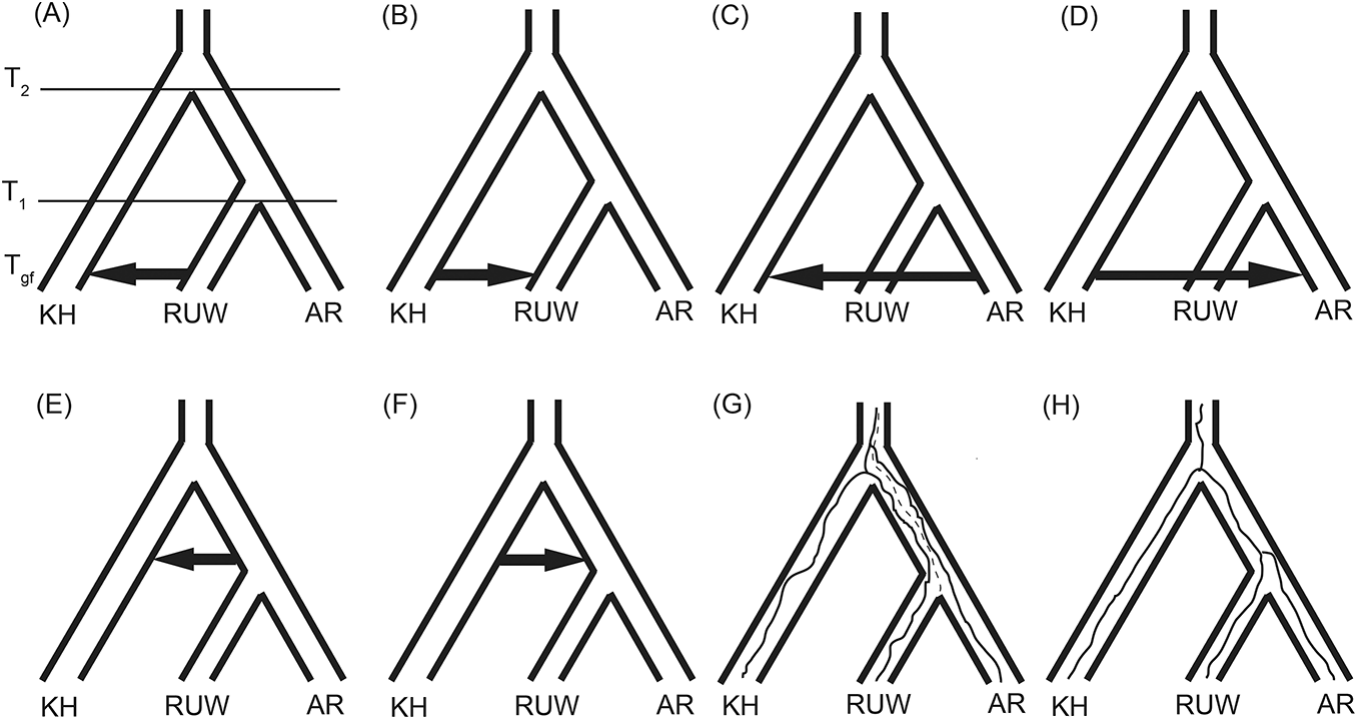
Schematic representation of the eight models (scenarios for evolutionary history) tested in this study. (A–F) Divergence with directional admixture between Kenyan Highlands, Albertine Rift, and Ruwenzori Mts, (G) ancestral population structure, and (H) strict divergence.

To distinguish the admixture events inferred in TreeMix from retention of ancestral polymorphisms, we chose the coalescent model-based method of Lohse et al. [22] that uses a full likelihood approach to explicitly test 8 competing models of divergence, admixture, and ancestral structure among AR and KH shrew and mouse species (Fig 6). This approach identified the model involving admixture between KH species (*H*. *endorobae* and *S*. *mundus*) and AR species (*H*. *vulcanorum*, *H*. *denniae* and *S*. *vulcanorum*, *S*. *granti*) to have the highest ML scores (Table 3). However, the likelihood ratio test for the best-supported shrew model (admixture from *S*. *mundus* into *S*. *vulcanorum* and *S*. *granti*) was not significantly different from a strict divergence model that accounted for ancestral polymorphism (*χ* ^*2*^ = 3.46, df = 2, *P* = 0.177). In *Hylomyscus*, the best-supported model involved admixture from *H*. *endorobae* into *H*. *denniae* and *H*. *vulcanorum* (*χ* ^*2*^ = 42.8, df = 2, *P* < 0.001) yet the timing of admixture for that model was indistinguishable from the divergence time estimate between *H*. *denniae* and *H*. *vulcanorum* such that it collapses to a strict divergence model (Table 3).

**Table 3:** Difference in support between best fit model and alternative models of admixture, divergence and ancestral structure with parameter estimates for all models.

Model description	ΔlnL M	ΔlnL S	*f* M	*f* S	*Θ* M	*Θ* S	*T*_gf_ M	*T*_gf_ S	*T*_1_ M	*T*_1_ S	*T*_2_ M	*T*_2_ S
Admixture from KH into AR	-19.4	-1.73	0.00	0.00	0.20	0.10	1.28	2.40	0.56	0.03	0.00	0.07
Admixture from AR into KH	-19.4	-1.73	1.00	0.00	0.20	0.10	1.84	1.12	0.00	1.31	0.03	0.07
Admixture from KH into RUW	-9.8	-1.38	0.07	0.02	0.19	0.09	0.64	0.00	1.31	2.51	0.03	0.10
Admixture from RUW into KH	-9.7	-1.38	0.07	0.02	0.19	0.09	0.63	0.00	1.32	2.51	0.03	0.10
Admixture from RUW+ AR into KH	-19.4	-1.73	1.00	1.00	0.20	0.10	0.00	0.07	1.84	2.43	0.16	0.43
Admixture from KH into RUW+AR	0	0	0.50	0.54	0.16	0.04	0.00	0.12	2.00	6.30	1.11	3.21
Ancestral structure	-21.4	-1.74	NA	NA	0.20	0.10	NA	NA	1.78	2.39	0.02	0.11
Strict divergence	-19.5	-1.73	NA	NA	0.20	0.10	NA	NA	1.81	2.42	0.00	0.07

M is abbreviation for mouse (*Hylomyscus*) and S for shrew (*Sylvisorex*). *f* is the admixture proportion, *Θ* is the scaled mutation rate, *T*_gf_ is the time of admixture, and *T*_1_ and *T*_2_ are the older and younger population split times, respectively.

**Fig 7:**
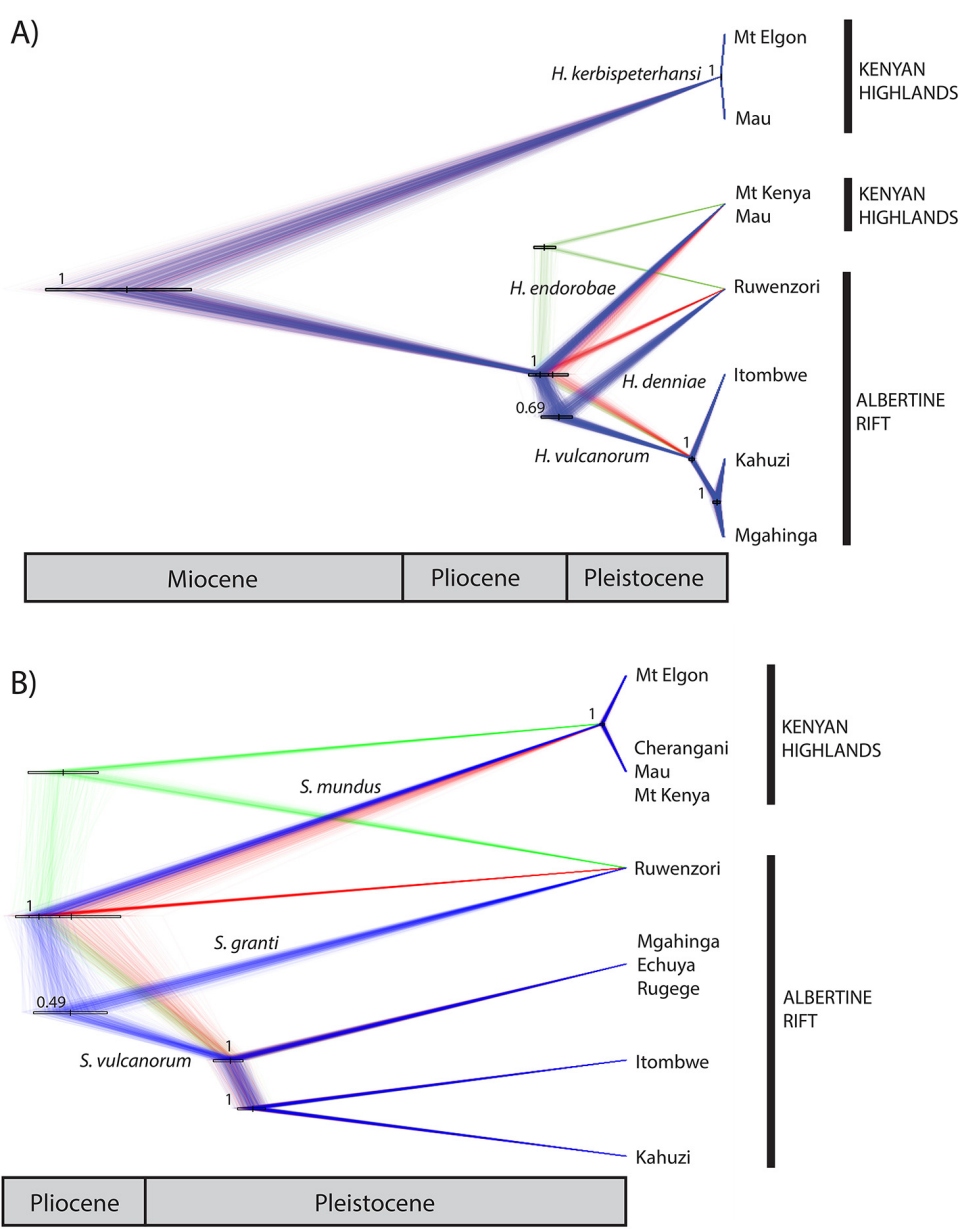
Maximum clade credibility population trees. Inferred for *Hylomyscus* (A) and *Sylvisorex* (B) using SNAPP and visualized using DensiTree. Branch lengths are a relative measure of substitutions per site. Posterior support values appear to the left of nodes. Divergence time estimates were calculated using a mutation rate of 1.0 x 10^−8^ [47,48] and upper and lower 95% confidence intervals are shown on the node bars. The median divergence times appear as vertical bars bisecting the 95% HPD bars.

Results from the species-tree phylogenetic analysis (SNAPP) were similar to previous estimates obtained in *BEAST using mtDNA and intron sequence data [6] with the following exceptions: in the *Hylomyscus* population tree *H*. *denniae* is supported as sister to a monophyletic *H*. *vulcanorum* clade, and all terminal branches are relatively short for intraspecific *Hylomyscus* populations (Fig 7). The *Sylvisorex* populations in western AR are supported as sister and have substantial geographic substructure, especially compared to *Hylomyscus*. Terminal branches among *S*. *vulcanorum* populations in the AR are significantly longer than those for KH populations of *Sylvisorex* and *Hylomyscus* populations from both regions.

## Discussion

This study used RAD-Seq to generate representational genomic data sampled from two co-distributed sets of populations of endemic Afromontane small mammals to test the hypothesis that AR populations are older and more persistently isolated than KH populations. To this end, we used the data and subsequent parameter estimates and model tests to compare with the predictions of greater genetic diversity, older intra-regional divergence times and greater population structuring in the AR regions for both species complexes. Our analyses are consistent with processes of long-term isolation within and among both species complexes, and we estimate parameters relevant to population relationships and demographic histories that were previously inferred from only one or a few independent loci [4,6,7]. Although our results support some key inferences from a previous study sampling the same *Hylomyscus* and *Sylvisorex* populations at 3–5 loci [6], inferences from these new data provide several points of departure and offer substantially more statistical confidence over the previous findings. In both species complexes, the data support the prediction that populations from the AR have a longer history of local isolation and persistence. This is consistent with the AR as an older, more topographically complex, and more climatically stable region, such that the longer persistence of suitable habitat throughout the cyclical climate fluctuations of the Pleistocene in the AR contributed to the region being a mammalian biodiversity hotspot. For *Hylomyscus* and *Sylvisorex* specifically, the AR may retain greater population structuring and intra-specific genetic diversity either as a result of having larger and more stable populations, or because it is also conforms to a meta-population with more demes having lower local extinction rates and/or lower migration rates.

These patterns of phylogenomic structuring within the AR support the long-standing hypothesis that the eastern margins of the Congo Basin, with the AR, supported refugia as old as the Miocene. This is further supported by patterns of endemism, species richness and discontinuities in the geographic distributions of mammals [52,53], birds [54–56], and palynological data [27]. Multiple phases of habitat connection and disconnection driven by climatic cycling coupled with the retention of paleoendemics in regions of refugial climatic stability have been hypothesized to explain the high species richness of the eastern Congo Basin lowlands and AR and also the distributions of biogeographic and phylogenetic relicts.

### Albertine Rift refugial stability with isolation

The timing of intraspecific population divergence time estimates provide evidence for long-term population persistence that may be correlated with regional climatic stability and deep intraspecific sub-regional isolation across the AR Highlands. Within *Hylomyscus*, the topology of the ML tree inferred in TreeMix under the assumption of no admixture supports longer term persistence and isolation among AR lineages compared to those from the KH. Terminal population branch lengths are significantly longer in the SNAPP phylogenies for AR endemic *H*. *vulcanorum* and *S*. *vulcanorum* compared to KH endemic *H*. *kerbispeterhansi* and *S*. *mundus*. This pattern is replicated to a lesser degree for the same taxa in the TreeMix graphs with migration (Figs 4 & 5). The conflicting support for post-divergence gene flow between AR and KH species based on two different analyses precludes drawing conclusions as to any relationship between sources of admixture and population stability and long-term isolation. However, the elevated heterozygosity of AR populations could be the result of larger effective population sizes, which along with the large degree of AR population structure is consistent with palaeo-climatological data that strongly supports the AR as a climatically stable forest refugia across multiple aridification intervals throughout the Plio-Pleistocene [27–29].

Within *Sylvisorex*, the well-supported AR lineages from the reticulating gene network, the topology of the ML tree inferred in TreeMix under the assumption of no admixture and the MCMC population tree topology inferred using SNAPP all support the hypothesis that AR populations are older and have been more persistently isolated than KH populations (Figs 6 and 7). The substructure apparent in the first two principal components of the genomic variation and the interspecific population clustering analysis additionally suggest long-term isolation of AR populations of *S*. *vulcanorum* (Fig 2). This is consistent with long-term maintenance of suitable habitat within multiple isolated AR refugia that coincide with major montane blocks (Fig 1).

### Kenyan Highland habitat instability and isolation from AR

Within the KH, populations of *Hylomyscus* and *Sylvisorex* exhibit the greatest genetic partitioning of Mt Elgon from populations to the east (*H*. *kerbispeterhansi F*_ST_ = 0.351, *S*. *mundus F*_ST_ = 0.295–0.310). This high level of partitioning between Mt Elgon and Mau populations was unexpected given the presence of lowland forest between these two montane forest blocks. This is particularly noteworthy when compared to the lower level of differentiation between Mau and Mt Kenya, which are separated by xeric non-forested Gregory Rift Valley habitat (*S*. *mundus F*_ST_ = 0.184). One possibility is that Mt Elgon and Mau populations are more stable than those in Mt Kenya. In this case, the Mt Kenya area could have been recolonized after late Pleistocene local extinction, whereas Mt Elgon and Mau populations persisted through multiple late-Pleistocene climate cycles thereby accumulating more inter-population genetic divergence with limited inter-population gene flow through the intervening lowland forest habitat.

Intraspecific population structure is also apparent within the KH for *H*. *kerbispeterhansi* and *S*. *mundus* based on population clustering methods. However, the timing of the divergence of Mt Elgon populations from Mau in *Hylomyscus*, and Mt Elgon from Cherangani + Mau + Mt Kenya in *Sylvisorex*, are much more recent than divergence times within the AR based on estimates of split times in the SNAPP population tree (Fig 7); these results are consistent with recent range-wide KH expansion. The two-fold higher levels of observed heterozygosity in populations of KH shrews from Mt Elgon as compared to populations in more eastern KH montane forests in both *Hylomyscus* and *Sylvisorex* (0.011 & 0.018 vs. 0.005 & 0.009, respectively) are consistent with Mt Elgon being a refugial center within the KH. Geologically, the extinct Mt Elgon volcano is much older than Mt Kenya in the East (Miocene vs. Late Pliocene-Early Pleistocene) although it does not possess more endemic mammal species than the Mt Kenya–Aberdare Mts ecosystem that is surrounded by a more arid matrix of dry woodland habitats [50,57]. Further phylogenetic studies of Mt Elgon’s fauna are needed to determine if older lineages have been retained compared to central Kenyan mountain regions. It is possible that greater climatic instability within the KH has prevented the persistent retention of suitable habitat at a metapopulation scale for both shrews and mice, thus eliminating patterns of long-term regional substructure.

Several lines of evidence support the pre-Pleistocene divergence and persistence of KH focal taxa across multiple glacial cycles with associated pulsed expansions and contractions of montane forest. Based on rough estimates of divergence times derived from population trees and reciprocal monophyly of the three endemic KH mouse and shrew species we sampled, there is little evidence for persistent connections between these two centers of Afromontane endemism. Data presented here also strongly supports the genetic isolation and deep divergence of Kenyan populations of *Sylvisorex granti mundus* from the Ruwenzori Mts endemic, *S*. *granti granti*. This is congruent with the recognition of *S*. *mundus* as a valid species. A recent revision of the *Hylomyscus denniae* complex similarly elevated *H*. *denniae endorobae* from Kenya to species. [50].

## Conclusions

All analyses strongly support the evolutionary independence of the three recognized species within the *Hylomyscus denniae* group (40, 47) and the recognition of *Sylvisorex mundus* as a valid species sister to *S*. *granti and S*. *vulcanorum*. This is consistent with an earlier phylogeographic study using far fewer loci (14). Also congruent with these earlier analyses are phylogenetic analyses presented here that show longer branch lengths among lineages within the Albertine Rift. In contrast, the large genomic dataset analysis provides several new insights. This includes a strong signal of historic and cryptic isolation of populations within both *Hylomyscus* and *Sylvisorex*. This is apparent for montane populations from the Itombwe Massif and for two additional *S*. *vulcanorum* lineages from the AR. Multiple analyses support the AR as harboring a substantial number of cryptic lineages and this is consistent with a long regional history of isolation and environmental stability. These data also support a potential response of populations within the *Sylvisorex granti* group across the Albertine Rift region to climatic shifts during the Plio-Pleistocene that periodically reduced and fragmented suitable habitat. This climatic periodicity is thus congruent with earlier inferences based on multi-locus species trees and paleo-distribution models, but the strength of the response indicated by this study is much more evident. Finally, our results also strongly support the historical isolation and evolutionary independence of the Itombwe Massif populations from other Albertine Rift mountain block populations. This is consistent with previous studies that found this sub-region to house the highest species diversity within the Albertine Rift [58,59].

## Supporting Information

S1 Appendix NCBI SRA accession numbers, Field Museum of Natural History voucher accession numbers, and locality information for all study specimens. (XLSX)
